# Issues in learning an ontology from text

**DOI:** 10.1186/1471-2105-10-S5-S1

**Published:** 2009-05-06

**Authors:** Christopher Brewster, Simon Jupp, Joanne Luciano, David Shotton, Robert D Stevens, Ziqi Zhang

**Affiliations:** 1 Aston Business School, Aston University, Aston Triangle, Birmingham, B4 7ET, UK; 2 School of Computer Science, Manchester University, Oxford Road, Manchester, M13 9PL, UK; 3 Harvard Medical School, Avenue Louis Pasteur, Boston, MA 02115, USA; 4 Image Bioinformatics Research Group, Department of Zoology, South Parks Road, Oxford, OX1 3PS, UK; 5 Department of Computer Science, University of Sheffield, Sheffield, S1 4DP, UK

## Abstract

Ontology construction for any domain is a labour intensive and complex process. Any methodology that can reduce the cost and increase efficiency has the potential to make a major impact in the life sciences. This paper describes an experiment in ontology construction from text for the animal behaviour domain. Our objective was to see how much could be done in a simple and relatively rapid manner using a corpus of journal papers. We used a sequence of pre-existing text processing steps, and here describe the different choices made to clean the input, to derive a set of terms and to structure those terms in a number of hierarchies. We describe some of the challenges, especially that of focusing the ontology appropriately given a starting point of a heterogeneous corpus.

Using mainly automated techniques, we were able to construct an 18055 term ontology-like structure with 73% recall of animal behaviour terms, but a precision of only 26%. We were able to clean unwanted terms from the nascent ontology using lexico-syntactic patterns that tested the validity of term inclusion within the ontology. We used the same technique to test for subsumption relationships between the remaining terms to add structure to the initially broad and shallow structure we generated. All outputs are available at .

We present a systematic method for the initial steps of ontology or structured vocabulary construction for scientific domains that requires limited human effort and can make a contribution both to ontology learning and maintenance. The method is useful both for the exploration of a scientific domain and as a stepping stone towards formally rigourous ontologies. The filtering of recognised terms from a heterogeneous corpus to focus upon those that are the topic of the ontology is identified to be one of the main challenges for research in ontology learning.

## Introduction

Ontology construction and maintenance are both labour intensive tasks. They present major challenges for any user community seeking to use sophisticated knowledge management tools. One traditional perspective is that once the ontology is built the task is complete, and thus ontology engineers have tended not to worry about the effort required for the building task. The reality of ontology development is, however, significantly different. The life sciences are a large, diverse and rapidly changing domain; an ontology will never be complete, but will change as understanding of the domain changes. For some large, widely used ontologies, such as the Gene Ontology [1], a manual approach is effective, even if expensive in terms of human effort, time and money. For small communities of interest in the sciences with limited resources, such manual approaches are unrealistic. This problem is all the more acute as research in many areas, including the life sciences, is moving to an industrialised e-science paradigm, with the rapid generation of large volumes of new data requiring machine-readable annotations. In such a context, we explore the use of techniques developed to learn ontologies from text to reduce effort in the early stages of ontology development.

The work presented in this paper concerns the semi-automatic construction of an ontology for the animal behaviour domain. The animal behaviour community has recognised the need for an ontology in order to annotate a number of data sets and provide a standard vocabulary. These data sets include texts, images and video collections. Existing controlled vocabularies or taxonomies have been used where relevant (such as Dublin Core [2], UBIO [3], and Ecoregion [4]), but a suitable ontology for concepts specific to animal behaviour are missing.

**Figure 1:**
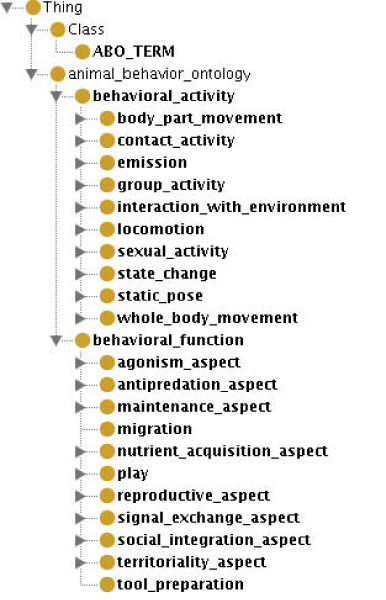
**Animal Behaviour Ontology (top level)**.

In two workshops in April 2004 and September 2005, an initial effort was made to construct an ontology for the purposes of applying annotations to these data sets. The current Animal Behaviour Ontology (ABO) has 339 classes and the top level structure is shown in Figure 1. Further information on ABO is available from [5] and [6].

While considerable effort has already gone into the construction of this prototype Animal Behaviour Ontology, its limited size raises the important question as to whether it is more appropriate to slowly build an ontology entirely by hand, and have its potential expansion led by user demand, or whether to rapidly build a much larger ontology based on the application of a variety of text processing methods, and then tidy or clean the output. With community engagement comes growth, but there is the need to stimulate engagement through some initial critical mass of useful ontology. The former is the standard approach and has been used successfully in cases such as the Gene Ontology, but becomes more challenging as the size and complexity of the ontology increases.

While much has been written about automatic ontology learning, most such work has been undertaken in non-biological domains, or in rather abstract contexts [7-9]. Although such research is called 'ontology learning', in reality, given the limitations of Natural Language Processing, the outputs have been structured vocabularies organised in taxonomic hierarchies. This might be considered a major defect if it were not that a) most ontologies are used for labelling/annotation purposes rather than for computational inference, and b) a hierarchically structured vocabulary based on the actual terminology used by a community is a major step towards the creation of a more ontologically formal, semantically strict ontology. In our view, the construction of formal ontologies of the type needed for driving semantic applications should be considered to involve a significant manual step following any automated process [10,11]. A small initial manual step can also be used to bootstrap the automated expansion of a nascent ontology [12], but the output of the automated stage will still need considerable manual input. In the research reported here, we chose to see what kind of basic ontology-like structure we could 'learn' from a corpus of text in the context of limited human resources. We approached the challenge as being one to construct a controlled or structured vocabulary as quickly as possible (in terms of actual human time spent, rather than computer time), with minimal effort, and then allow subsequent efforts to clean up the output of this exercise. At one level, we have tried to assess how much effort is worth investing and what is the balance of cost and benefit. A greater understanding of the most effective methods will in the longer term not only facilitate the creation of useful ontologies with limited resources, but will also facilitate the growing issue of maintenance and upkeep of ontologies as a whole. In this work we have attempted no novel natural language processing techniques in ontology learning from text. Rather, we have explored how to use existing techniques within the context we have outlined and what issues these raise for ontology development.

## Methodology

The process we adopted to explore whether we could generate an ontology of animal behaviour with relatively low human effort can be summarised as follows:

• Prepare a corpus of text from a research journal on animal behaviour;

• Apply automatic term recognition techniques to that corpus;

• Extract animal behaviour terms from the general set of terms;

• Assemble those terms into an ontology-like taxonomic structure.

These steps are detailed more fully below.

### The data set

It has been argued elsewhere that an effective way to build representative ontologies for a given domain is through the use of text corpora [13]. In our case we were given access to a corpus of journal articles from *Animal Behaviour*, published by Elsevier. This consisted of Volumes 71 (2006) to Volume 74 (2007), containing 623 separate articles. We were given access to text, PDF and XML versions, together with a corresponding DTD. We used the XML version for the procedures that are described below.

### From text to Ontology

1 Clean text was extracted from the XML files. Using the information from the structured markup, we excluded all author names, affiliations and addresses, acknowledgements, and all bibliographic information, except for the titles of the cited papers. This is a first measure used to exclude unwanted material from the ontology building process. The Animal Behaviour journal defaults to British English – according to the author instructions 'Use British spelling and grammar conventions throughout, except in non-British quotations and references [14], consequently our methodology does not need to take this issue in to account. With a more general corpus, however, spelling would have to be normalised and this would be performed using a dictionary based look up.

**Figure 2:**
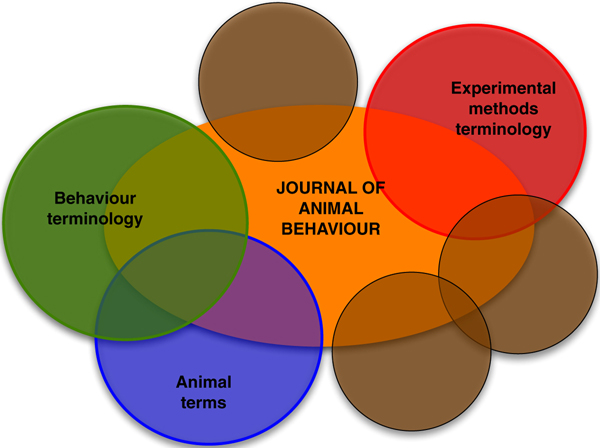
**Aspects of a corpus (brown circles represent as yet unspecified ontologies or sets of terms)**.

2 A number of stop word lists and gazetteers were used to further remove noise from the data. We excluded author names as noted above and also, through the use of a number of gazetteers, we excluded various types of locations, and the first and last names of other people. Animal names were excluded using a list of 892 animals derived from the LDOCE (The Longman Dictionary of Contemporary English. Our thanks to Louise Guthrie for providing this.). Such terms are the subject of different ontologies (for example one of animals, or the Ontology for Biomedical Investigation (OBI) [15] that include experimental and data analysis methods. Any journal corpus will cover many aspects of the scientific domain (see Figure 2) and thus one aim of ontology learning from such sources is to focus the ontology on a particular topic. This is not to say, for example, that animals and animal behaviour are unrelated, but that they belong in different ontologies and should be related compositionally. Such other ontologies are often the subject area of the OBO Foundry [16]).

3 A lemmatizer was used to increase coverage [17]. In some cases this generated some noise due to imperfections in the lemmatizer, but over all it reduced data sparsity (i.e. by bringing together different forms of the same word, the number of instances of any given word type is increased).

4 Five different term extraction algorithms were applied as described in [18]. The chosen algorithms were ones that selected both single and multi-word terms, as desirable technical terms are of both sorts. The algorithms were applied to each subsection of the journal article as well as to the whole. This allowed us to look at the terms from different sections of the articles (abstract, introduction, materials and methods, results, discussion and bibliography).

5a We then used a set of regular expressions to filter the candidate terms. This was constructed with human inspection of the term lists. The aim was to maximise recall leaving cleaning of the recovered terms until a later stage. A regular expression was constructed that looked for multi-word terms that ended in behaviour, display, construction, inspection, etc. The regular expression was designed to capture multi-word terms indicating animal behaviour (as well as the single 'head' words themselves). So for example, the regular expression attack$ would identify such multi-word terms as aggressive attack, successful attack, pathogenic attack, territory resident attack, infanticidal attack, simulated predator attack etc., only some of which relate to animal behaviour. The term lists were sorted on final word and a count made of word frequency. Any animal behaviour term occurring four times or more as a final word was added to the regular expression. The rationale was to balance recovery against ease of simply adding the term manually to the growing ontology. So, 'x inspection', 'y inspection', 'z inspection', etc. occurring four times or more would mean the element ' inspection$' would be added to the regular expression. It also included some generic regular expressions looking for terms that, for example, ended in -ing and -ism. This was to capture the large number of gerunds such as 'running', 'hunting', 'grooming', etc. and similar widely used patterns of morphological derivation. This will have obvious implications for precision, but our emphasis was on recall in order to have as many useful terms in the ontology as possible. The regular expression used is as shown in Table 1.

**Table 1:** The regular expression used in Step 5a.

defence$ | attack$ | behaviour$ |	preference$ | discrimination$ |
choice$ | selection$ | ing$ |	attraction$ | grunt$ | reduction$ |
care$ | conflict$ | aggression$ |	chase$ | aggregation$ | alarm$ |
ism$ | competition$ | recognition$ |	movement$ | investment$ | urination$ |
skill$ | ship$ | copulation$ |	submission | invitation$ | expulsion$ |
play$ | flight$ | flip$ | response$ |	motion$ | mimicry$ | release$ |
avoidance$ | fidelity$ | courtship$ |	icide$ | fight$ | rut$ |
inspection$ | intrusion$ | activity$ |	coercion$ | construction$ | flight$ |
reactivity$ | communication$ | attendance$ |	solicitation$ | search$ | appeasement$ |
igration$ | harassment$ | contest$ |	mimicry$ | protection$ | submission$ |
interference$ | foraging$ | polyandry$ |	preparation$ | vocalization$ | vocalisation$ |
predation$ | call$ | bob$ |	incubation$ | insemination$ | concealment$ |
intrusion$ | tactic$ | strategy$ |	evasion$ | nod$ | call$ |
attempt$ | trill$ | whistle$ |	trill$ | song

6a There are a number of methods that can take a set of terms and identify ontological (taxonomic) relations between them [7,8]. Most methods suffer from low recall. So in our approach we chose to apply to the output from 5a the method that literature showed to have the highest recall, namely 'string inclusion'. This means that a term A B***IS_A ***B, and a term A B C***IS_A ***B C, and also that term B C***IS_A ***C. The resulting ontology was saved in the Web Ontology Language (OWL) [19].

7a In an attempt to clean the non-animal behaviour terms from the ontology, the top level terms from the resultant ontology (i.e. the immediate descendants of the root) were then filtered as to whether or not they were actually kinds of behaviour. A technique used extensively in the ontology learning community is that of using lexico-syntactic patterns (or Hearst patterns [20]) to either learn or test for a candidate ontological relation [8]. In this case, we wanted to test each top level term in each ontology as to whether it was a kind of behaviour or one of its synonyms activity, conduct and action. Thus we automatically constructed lexico-syntactic phrases and queried the Web for their occurrence. Example phrases included "behaviours such as biting" (found) or "behaviours such as dimorphism" (not found). A list of the phrases used is provided in Table 2. The web was queried using the Yahoo BOSS web service [21]. If a query phrase was found to have a hit, this was taken as a legitimate term (no matter the hit count) and if there were no hits this term was considered not to be a behaviour term and thus excluded. The exclusion of a top level term also resulted in the exclusion of children of that term.

**Table 2:** Lexico-syntactic phrases used in Step 7a and 7b.

< Child singular > or other < Parent plural >
< Child plural > and other < Parent plural >
< Child plural > or other < Parent plural >
< Child singular > is a type of < Parent singular >
< Child singular >, a type of < Parent singular >
< Child singular > and other < Parent plural >
< Parent plural > such as < Child plural >
< Child singular > is a kind of < Parent singular >
< Child singular >, a kind of < Parent singular >

8a In order to provide a more effective hierarchical structure, we used a method similar to the one used in Step 7a. For each top level term, lexico-syntactic phrases were constructed to test whether it has an ***IS_A ***relationship with every other top level term. Thus for example, the first top level term (alphabetically) in the output of 7a is absenteeism and the second is accepting. So lexico-syntactic phrases are constructed to test whether there is on the Web any evidence of an ***IS_A ***relationship, e.g. "absenteeism is a type of accepting" (0 counts) and "accepting is a kind of absenteeism" (0 counts). Where we do get a count, this is used as evidence of an ontological (***IS_A***) relationship. This procedure involves testing every possible pair of top level terms against 9 different lexico-syntactic structures, resulting in nearly 32M queries (again using the Yahoo BOSS web service). A more detailed description of the theory behind the methodology is provided in [22,23].

5b The step described in 5a involved specific domain knowledge. To have an alternative procedure that involves no domain knowledge, we used a voting algorithm to rank the terms and weight them for distribution across the corpus. This was calculated by taking the mean rank for each term and multiplying by the document frequency as shown in the following formulae:

(1) $$\bar{rk}(t_{j})=\frac{1}{n}\Sigma_{i=1}^{n}rk_{i}(t_{j})$$

(2) $$RK(t_{j})=\bar{rk}(t_{j})d(t_{j})$$

where *rk*_*i*_(*t*_*j*_) is the rank of term *t*_*j *_using term recognition algorithm *i*, *d*(*t*_*j*_) is the number of documents in the corpus in which term *t*_*j *_occurs, and *RK*(*t*_*j*_) is the final overall ranking for term *t*_*j*_. A more detailed description and context is provided in [18]. From the resulting rankings, the top *n *terms were selected for the subsequent steps (to parallel those extracted by the regular expression in 5a), where in this case *n *= 13, 755.

6b The same method described in 6a was separately applied to the output of 5b.

7b The method described in 7a applied to the output of 6a was separately applied to the output of 6b.

8b The method described in 8a was separately applied to the output of 7b.

**Figure 3:**
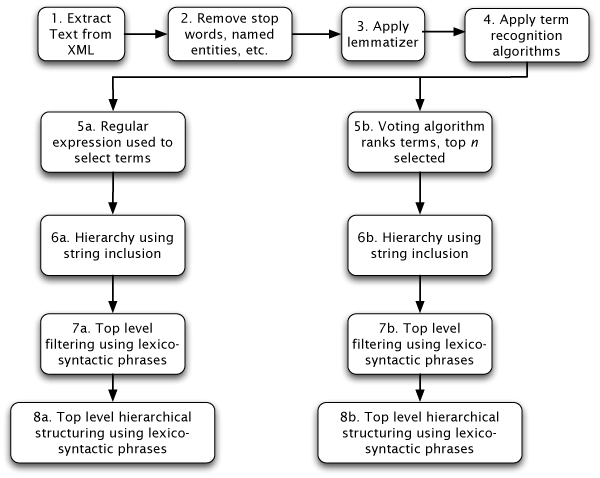
**The processing steps undertaken**.

These steps are summarised in the flowchart shown in Figure 3.

### Evaluation steps

We undertook the following steps to evaluate the output of our procedures:

#### 1. Excluded terms

A sample comprising about 4% of the terms (3140 terms) excluded by Step 5a was evaluated by a biologist (Shotton) to determine the proportion of 'correct' terms incorrectly excluded.

#### 2. Selected terms

In order to calculate the precision of the terms selected by Step 5a, a program was created to present to the biologist at random a sample of about 15% of the terms from that set (2070 terms). He then specified whether each presented term was a bona fide animal behaviour term or not, resulting in two files of valid and invalid terms. A similar set of 2287 terms selected by the voting algorithm (Step 5b) were also evaluated in the same way.

#### 3. Final subsumption pairs

In order to evaluate the quality of the subsumption pairs (parent-child pairs) in the output of 8a, we presented the biologist with a small random sample of such pairs and asked him to state whether (a) the child term was a bona fide animal behaviour term, (b) whether parent term was a bona fide animal behaviour term, and (c) whether the subsumption pair was a valid ontological pair.

#### 4. Qualitative evaluation

**Figure 4:**
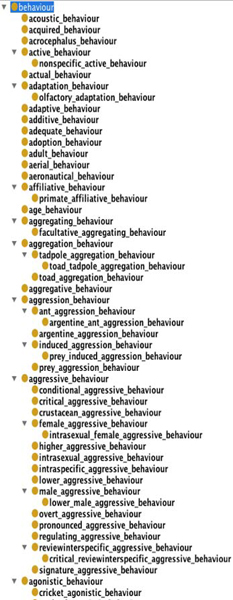
**Screen-shot of part of the sub-tree concerning **behaviour **from the output of Step 8a**.

**Figure 5:**
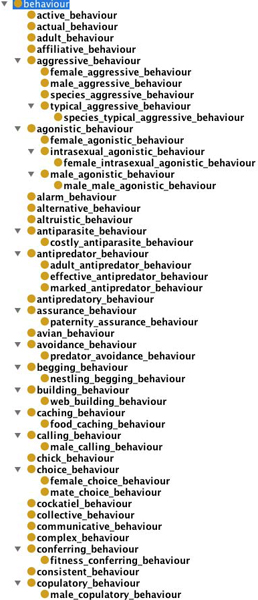
**Screen-shot of part of the sub-tree concerning **behaviour **from the output of Step 7b**.

**Figure 6:**
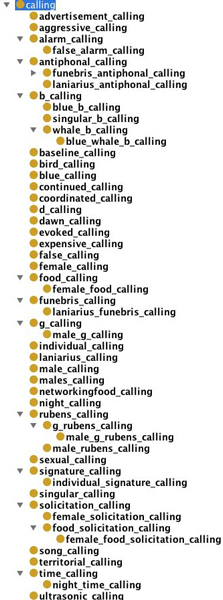
**Screen-shot of part of the sub-tree concerning **calling **from output of Step 8a**.

**Figure 7:**
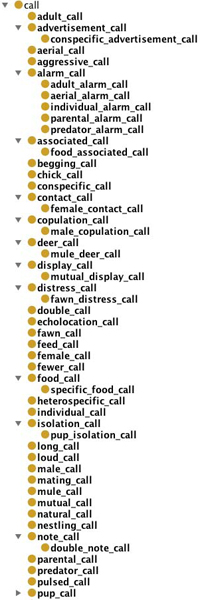
**Screen-shot of part of the sub-tree concerning **call **from the output of Step 7b**.

The ontologies created in Step 8a (partially shown in Figure 4 and in Step 8b (partially shown in Figure 5) were evaluated by our domain expert (Shotton), who inspected the ontology for biological correctness and usefulness at the initiation of an ontology building process. In addition to comparing Figures 4 and 5, we invite the reader to compare the generated subtrees for calling (from Step 8a, Figure 6) and for call (Step 8b; Figure 7).

## Results

### Term extraction

A total of 98 435 terms were extracted from the whole corpus of 2.2 million words in Steps 2–4 (Table 3). From this, the regular expression was used to extract 13 755 terms (Step 5a). In a parallel manner, the top 13 755 terms were selected from the output of the term voting algorithm (Step 5b). In both Steps 5a and 5b, the terms recovered included both animal behaviour terms and a large number of non-animal behaviour terms. The regular expression was designed to capture terms such as begging, foraging, dancing, grooming, burrowing and mating, but due to its crudity it also picked up non-behavioural terms with similar endings, such as bunting, herring, dichromatism and dimorphism. A sample of the terms picked out in Step 5a is given in Table 3. Some of these terms reflect low level errors in handling spaces, etc.

**Table 3:** Random sample of terms extracted by Step 5a.

colony fissioning	active territory defence
high investment	matching neighbour song
greater reduction	soldier bug predation
major force favouring	stranger song
eavesdropping and mate choice	severe matriline based aggression
mormoniella and pachycrepoideuspolyandry	dashing
threat grunt	acid metabolism
total attraction	taeniopygia guttatasong
location and spacing	acoustic response
orienting response	prior winning
memorize heterospecific song	daytime rest and sleep behaviour
diet selection behaviour	sexual and courtship behaviour
mass rearing	xiphophorusfemale preference
diurnal activity	utetheisa ornatrixsite dependent aggression
locomotor behaviour	
noise interference	

### Term distribution

As part of Step 2, we also examined which sections of the research articles were most profitable for the retrieval of terms. Table 4 shows the number of words, terms retrieved and animal behaviour terms retrieved by the regular expression from these different sections. We tested for a statistical difference from background frequency of occurrence of animal behaviour terms using a G-test. This shows that:

• The Introduction and Discussion do not differ significantly from the background frequency of animal behaviour terms (*P *= 0.315; *P *= 0.242).

• The Materials & Methods and Results sections have significantly fewer animal behaviour terms than the background frequency (*P *= 1.04 × 10^-9^; *P *= 1.99 × 10^-13^).

• The Abstract and Bibliography have significantly more animal behaviour terms as a proportion of total terms than average (*P *= 6.03 × 10^-10^; *P *= 2.69 × 10^-128^). The bibliography, which consists only of the titles of the papers cited in each article, was particularly enriched with animal behaviour terms.

**Table 4:** The number of terms and animal behaviour terms retrieved from each section of the corpus.

**Section**	**Total words**	**Total terms**	**Animal Behaviour Terms**	**Proportion of Behaviour Terms**
Title and Abstract	131132	13463	2031	0.15
Introduction	525841	36932	4915	0.13
Materials and Methods	646675	13611	1535	0.11
Results	323196	19504	2197	0.11
Discussion	260168	20170	2683	0.13
Bibliography (titles only)	318678	39115	6336	0.16
Total (non unique)	2205690	142795	19697	
				

Total unique terms		98435		
Total unique animal behaviour terms		13755		
Proportion of animal behaviour terms		0.14		

### Ontology construction

The ontology produced from the 13,755 terms selected by the regular expression in Step 5a by application of the string inclusion method in Step 6a resulted in an artefact of 18 171 classes, of which 1 294 classes are top level (i.e. direct children of owl:Thing). Similarly the ontology produced by Step 6b. from the 13 755 terms selected by the voting algorithm in Step 5b. resulted in an artefact of 14 251 classes, of which 2 535 classes were top level (Table 5). Both ontologies are broad and shallow, but the ontology produced from the voting algorithm selected terms has many more top-level terms.

**Table 5:** Data sets at each step 1 – 8

Step	1	2,3	4	5a	5b	6a	6b	7a	7b	8a	8b
Number of articles	623										
Number of noun phrases		135026									
Number of terms			98435								
Number of terms selected				13755	13755						
Number of classes						18171	14251	18055	12383	18055	12383
Subclass axioms						16877	11716	16876	10497	17393	12326
Top level clases						1294	2535	1179	1886	662	58
Maximum Depth						5	5	5	5	8	13
Average Depth						2.4	2.1	2.4	2.2	3.1	1.4
Maximum span						1294	2535	1179	1886	778	557
Average Span						1.7	1.8	1.7	1.8	1.7	2.2

The filtering process described in Step 7a resulted in 115 top level terms together with their descendant sub-classes being removed, leaving 1 179 immediate descendants of owl:Thing. Top level classes that were filtered out by this method included terms such as stocking, referencing, holding, attraction, time, schooling, movement, pacing, defending, smashing, loading, matricide. Unfortunately, some of these removed terms (holding, schooling, pacing, defending, matricide) are in fact *bona fide *animal behaviour terms. The total number of classes in this filtered ontology was 18 055.

The parallel process in 7b resulted in 649 top level classes being removed, together with the corresponding sub-classes, leaving 12 383 classes in total, and 1 886 top level classes.

The restructuring of top level terms that occurred in Step 8a resulted in 689 top level terms, without changing the total number of classes (18 055). Sample output of 8a and 7b for the sub-trees concerning behaviour and call/calling is shown in Figures 4 and 5, and in Figures 6 and 7, respectively. The ontologies mentioned here are available on the web site containing the Supplementary Information accompanying this paper 

### Evaluation results

1. In order to evaluate Step 5a, a random sample of 3 140 terms, taken from the 84 680 terms excluded from the original 98 435 terms by use of the regular expression in Step 5a, were manually inspected. From these 49 terms (7 verbs and 42 nouns), 1.56% of the sample, were identified as *bona fide *animal behaviour terms, including forage, strike, secretion, ejaculate, higher frequency yodel, and female purring sound. This low number of wrongly excluded terms shows that our approach has a Negative Predictive Value of ≈ 98%(= 3091/3140). Assuming that this sample was representative of the whole, this indicates that some 1321 *bona fide *animal behaviour terms (1.56% of 84,680) originally present in the corpus were wrongly excluded by Step 5a (Table 6).

**Table 6:** Results from Steps 1-5a and 5b, and the Evaluation Steps 1 and 2.

	Regular expression method		Term voting method	
TOTAL terms	98435	100%	98435	100%
Selected set	13755	14%	13755	14%
Excluded set	84680	86%	84680	86%
Sample of excluded	3140	100%		
Wrong (false negative)	49	1.6%		
Correct (true negative)	3091	98.4%		
Proportionate number of *bona fide *terms in excluded set	1321			
Sample of included	2070	100%	2287	100%
Wrong (false positive)	1538	74.3%	1974	86.3%
Correct (true positive)	532	25.7%	313	13.7%
Probable number of *bona fide *terms in selected set	3535		1883	
**Recall**	0.728			
**Precision**	0.257		0.137	

2. Of the terms 13,755 terms recovered by the use of the regular expression in Step 5a, a total of 2 070 terms (just over 15% of the total) were randomly selected and then evaluated manually to see what proportion were valid animal behaviour terms. 532 of these terms (25.7%) were deemed relevant to the animal behaviour domain. Assuming that this sample was representative of the whole, this indicates that some 3 535 real animal behaviour terms (25.7% of 13 755) exist within the total pool of 13,755 extracted terms. (Table 6).

Thus we estimate that the original 98 435 terms contained a total of some 4 856 animal behaviour terms (1321 excluded plus 3 535 included), 3 535 of which were correctly extracted by the regular expression algorithm used in Step 5a. This gives an extraction recall of 72.8% (3 535/4 856), and an extraction precision of 25.7% (3535/13,755) at Step 5a in our processing (Table 6).

3. Of the terms selected using the top *n *as a result of applying the voting algorithm (Step 5b), a total of 2 287 were manually evaluated. Of these 313 (13.6%) were considered *bona fide *animal behaviour terms. Assuming that this sample was representative of the whole, this indicates that some 1 883 real animal behaviour terms (13.6% of 13 755) exist within the total pool of 13 755 extracted terms, with an extraction precision of 13.6% at Step 5b (Table 6).

4. For the evaluation of the subsumption pairs present in the ontology created at Step 8a, 204 pairs were presented to the biologist, and 57 were found to be valid, giving a precision of 27.9% (similar to the term precision). Full details are presented in Table 7. Examples of *bona fide *subsumption pairs include host finding *IS_A *finding, size matching *IS_A *matching, attack pursuit behaviour *IS_A *pursuit behaviour. Examples of invalid pairs include extensive plugging *IS_A *plugging, structure infant handling *IS_A *infant handling, bisonvasopressin grooming *IS_A *grooming, raven behaviour *IS_A *behaviour, dependent averaging *IS_A *averaging. Note that a subsumption pair could be invalid either because it included a term which was not a *bona fide *animal behaviour term, or because the ontological relationship was incorrect. Terms involving animal names (e.g. raven), or referring to age, size or status were excluded as *bona fide *child terms.

**Table 7:** Results of the evaluation the subsumption pairs in the output of Step 8a.

Number of terms sampled	408
Number of valid terms	198
Number of incorrect terms	210
Term precision on this sample	0.49
Number of subsumption pairs in this sample	204
Number of valid subsumption pairs in this sample	57
Number of incorrect subsumption pairs in this sample	147
Precision of the subsumption pairs in this sample	0.28

### Ontology comparison

#### 1. Comparison with ABO

A comparison of the ABO ontology with the ones automatically generated in Steps 8a and 7b reveals an overlap of only 84 terms. The vocabulary of the ABO is severely restricted, compared to the automatically generated ones. For example, under sound_production there are only 11 terms in ABO, while in Ontology 7b, there are 74 terms under the top level class call alone, and in Ontology 8a there are 56 terms under the class calling, with further relevant sound_production terms under the classes song and singing.

#### 2. Comparison of the two automatically generated ontologies

A comparison of the two automatically generated ontologies 8a and 7b reveals that both cover a rich and relevant set of terms from a slightly different perspective. For example, because we filtered out top level terms (at Step 7a) which were **not **behaviour, activity, action, only the term calling appears in 8a with the above mention 56 sub-class terms including advertisement calling, dawn calling, male calling, etc. Ontology 7b includes the top level terms call, caller, and calling, with the majority of sub-class terms under call, including adult call, aggressive call, click call, display call and mating call.

To study the differences between the two classes of ontologies produced by our automated system, our biologist (Shotton) analysed 200 top-level classes from each ontology, the first 100 in the first half of the alphabet starting from A, and the first 100 in the second half starting from N.

(Since Ontology 8b contained only 58 top-level terms, of which only 20 were relevant to animal behaviour, the subsequent analysis was undertaken with the preceding ontology, Ontology 7b.) In Ontology 7b, these 200 classes included terms from abandonment to assignment and from na to paternity. Of these, 82 class names (41% of the top level terms) clearly relate in some way to animal behaviour, collapsing into 61 concepts if synonyms are equated. Only five of the relevant terms have identical or equivalent terms in Ontology 8a; thus 94% of these relevant terms are unique to this ontology (Table 8). Generalizing over the entire ontology of 1 886 top level classes, this equates to about 773 relevant classes, of which about 727 are unique to the ontology created by the term frequency voting method.

**Table 8:** Data from human evaluation of Ontologies 8a and 7b.

	Ontology 8a – Regex		Ontology 7b – Voting	
Total number of top-level terms	662		1886	
Number of sampled top-level terms	200	30.2% of total	200	10.6% of total
Number of sampled top-level terms relevant to animal behaviour	84	42% of sample	82	41% of sample
Proportionate number of top- level animal behaviour terms in the whole ontology	278		773	
Number of sampled top-level an imal behaviour terms not found in the other ontology	44	22% of sample	77	38.5% of sample
Proportionate number of top- level animal behaviour terms in the whole ontology not found in the other ontology	145		727	

In ontology 8a, which had fewer top-level classes overall (662), these 200 classes included terms from activity to complexlearning and from nepotism to remating. Of these, 84 class names (42% of the top level terms) clearly relate in some way to animal behaviour, with only one pair (behavior and behaviour) being synonyms. As stated above, only 5 of these terms related to the selected terms from ontology 7b. However this is not a fair comparison: since the number of top-level terms in ontology 8a is fewer than in 7b, the selected 200 covered a larger alphabetical range. Our biologist thus looked for the number of behaviour-relevant terms from 8a that had equivalents anywhere the 7b ontology, and found that 40 terms of the behaviour-relevant terms from 8a (48%) have identical or equivalent terms in Ontology 7b. Thus 52% of the relevant terms are unique to ontology 8a (Table 8).

Generalising over the entire ontology of 662 top level classes, this equates to about 278 relevant classes, of which about 145 are unique to the ontology created by the regular expression method.

Thus each method of term extraction used on the corpus is effective and complementary to the other, both being very good at producing lots of raw ontology-building material.

### An analysis of the hierarchies produced

**Figure 8:**
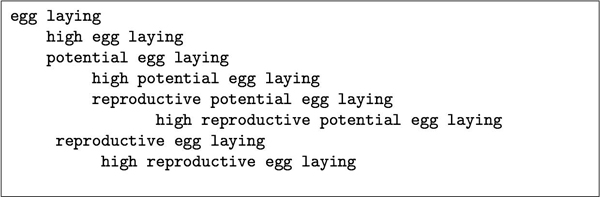
**A sample subsumption hierarchy generated from the phrase** "high reproductive potential egg laying" **using the string inclusion method**.

The string inclusion method of creating subsumption hierarchy is successful in places and unsuccessful in others. For example, a simple splitting of the term ABC will often provide a sensible hierarchy (e.g. intraspecific aggressive behaviour, stereotypic wire gnawing, competitive scent marking, or compensatory dietary selection). In contrast, with the term high reproductive potential egg laying, this simple approach leads to nonsense (Figure 8). In this case, the sub-term reproductive potential should be taken as one morphological unit and egg laying should be a second morphological unit. There are NLP techniques such as dependency parsing, bigram probability modelling, and named entity recognition (of species names) which can be applied to reduce the types of error shown in Figure 8. Without such an approach, the large amounts of noise seen in this simple example are inevitable.

The term defence appears in both ontologies. In Ontology 7b, there are only six sub-classes of this class, all legitimate, five of which are in common with Ontology 8a. Due to the term selection methods used in Steps 5b, Ontology 7b also has defender as a separate class. In Ontology 8a, there are 145 defence terms, nested 3 levels deep. Only about 27 of these are strictly relevant to animal behaviour, and putting them all into one ontology subclass mixes together things you would really want to keep separate. In the hand-built Animal Behaviour Ontology (ABO), there is no defence term at all, although there are 13 defence-related terms such as attack predator, feign death and mate guarding scattered across the ontology.

### The next step: Moving from automated results to a formal structured ontology

Using the terms recovered automatically, together with those already in the original ABO, our expert (Shotton) developed from scratch the start of an ontology describing various aspects of defence, using the technique of ontology normalization [24]. This contains the following separate ontology modules into which the mixed up terms can be segregated:

• behavioural action

• behavioural function (with the single subclass defence)

• defender (with five example subclasses added, e.g. parent, male mate)

• thing being protected (with six disjoint subclasses, for example conspecific animal and resource, each with their own subclasses)

• physical nature of defence (with subclasses active defence, e.g. mobbing, and passive defence, e.g. camouflage)

• social nature of defence (with four example subclasses)

• thing being defended against (with principal subclasses animal and environmental factor)

While use of the string inclusion method introduced a great deal of noise (Table 6), subsequent use of Steps 7 (filtering) and 8 (restructuring) organised the terms to an extent that makes the subsequent job of 'proper' ontology development easier for the expert. This was primarily achieved by grouping all the defence terms in one place, with a single word defence at the root acting as an index term; the hierarchies contained were not very useful.

This approach could clearly be generalised to other aspects of animal behaviour such as obtaining food or care for offspring. The consequence of that would be that the ontology module defender would be generalised to one entitled actor, that entitled thing being protected would be generalised to one entitled object of behavioural activity, that entitled thing being defended against would be generalised to proximate cause of behavioural activity, etc.

The primary benefit of the exercise is in extracting terms from the corpus that are of relevance to the domain. The simple organisation of the terms under root terms make these easier to find and use, but the large amount of noise is a severe distraction. Nevertheless, as our expert commented, 'it is much better than starting with a blank page'. Taken together, these observations leave us with the following tasks for future work:

• Improve filtering of terms for an increased focus;

• Improve morphological analysis of terms to improve string inclusion results.

• Decompose terms into separate modules for later composition.

### Effort in ontology building

The effort involved in building ontologies by hand is often hard to calculate, and it is difficult to obtain reliable figures. In Table 9, we show the estimated effort involved in building ontologies in which the authors of this paper have been personally involved, or have been able to ascertain from participants of manual ontology building exercises. It should be emphasised that such calculations are only indicative, as a great deal of the effort involved is not measurable, so the figures should be treated with caution. The figures presented in Table 9 were derived as follows (Note: We have assumed a working year of approximately 2000 hours):

• **BioPAX Level 1 – Metabolic Pathways Ontology **[25] The figures are based on the fact that a core group of 2–3 people worked on the project half time for 2.5 years (3 people × 0.5 time × 2000 hours × 2.5 years = 7500 hours), 10–11 people had bi-weekly conference calls lasting about 2 hours ((11 – 3) people × 20 weeks ×2.5 years × 2 hours = 800 hours), and in addition there were face to face meetings about 4–6 times per year lasting 2 days ((11 – 3) people × 5 meetings × 2.5 years × 2 days × 8 hours = 1600 hours). Total 9900 hours.

• **InfluenzO – the Influenza Ontology **[26] The effort involved here was approximately as follows: 1 person × 0.6 time × 1.5 years × 2000 hours = 1800 hours, plus 1/5 time for 0.5 years = 200 hours, plus 1 person × 1/5 time × 1.5 years ×2000 hours = 600 hours, plus 1 person × 0.1 time for 1.5 years × 2000 hours = 300 hours. Total hours equals 2,900.

• **OBI – Ontology for Biomedical Investigations **[15] The effort involved here was approximately as follows: 10 people involved over a 3 year period, with 2 workshops a year, with some people allowed to work full time on this including 4–5 core developers (2 full time people × 3 years × 2000 hours = 12,000 hours, plus 6 people × 3 years × 48 weeks × 2 hours = 1,720, plus 6 people × 6 workshops × 16 hours = 576). Total equals 14,296.

• **EFO – Experimental Factors Ontology **[27] The effort involved here was approximated as follows: 1 person × 10 days per month ×1 year = 960 hours, plus 1 person × 2 days per month × 1 year = 192 hours, plus 1 person × 3 days per month × 1 year = 288 hours. Total equals 1,440 hours.

• **ABO – Animal Behaviour Ontology **[5,6] The effort involved here was approximately as follows: First 2 day workshop × 20 people = 320 hours, plus Second 2 workshop × 10 people = 160 hours, plus 1 person × 20 days = 160 hours, plus 2 people × 10 days, all over a period of three years. Total equals 800 hours.

• **Normalisation of the CTO **[28] This task concerned the normalisation and transformation of the Cell Type Ontology into a highly axiomatised OWL ontology (undertaken as part of the Ontogenesis Project). Effort involved was approximately as follows: 10 people × 2 days = 160 hours, plus 12 people × 2 days = 176 hours, plus 2 people × 3 days = 48 hours, 3 weeks × 0.5 time × 1 person = 60 hours. Total equals 444 hours.

**Table 9:** No. of classes and effort involved in the production of a number of manually curated ontologies.

Ontology	No. of classes	Subsumption axioms	Duration	Effort in hours
ABO	305	303	3 years	800
Normalisation of CTO	1110	2823	8 months	444
EFO	1420	1873	1 year	1440
InfluenzO	269	223	2.5 years	2900
BioPAX L1	28	67	2.5 years	9900
OBI	1366	2016	3 years	14,296
GO	26894		10 years	> 160 000

The expense of ontology building is also indicated by the $16M spent on building the Gene Ontology (until *circa *2005, cited by [29]). The point about these figures is not their absolute accuracy but rather the scale and orders of magnitude involved. It is clear that manual ontology building involves a huge effort at great cost in labour and time. However sometimes, because the work is so incremental, the community may not fully appreciate the scale of that effort.

## Discussion

### Related work

There is a substantial literature in automated term recognition. In the majority of studies, linguistic processors (e.g. part of speech (POS) taggers, phrase chunkers) are used to filter out stop words and restrict candidate terms to nouns or noun phrases. In other studies, any n-gram sequences (sequences of words of various lengths where *n *is typically 2, 3 or 4) are selected as candidate terms. Early work was undertaken by Justeson and Katz [30], who identified syntactic patterns corresponding to typical technical terminology. Statistical measures are used to rank the candidate terms. These measures can be categorised into two kinds: measures of 'unithood', indicating the collocation strength of units that comprise a single term; and measures of 'termhood', indicating the association strength of a term to domain concepts [31]. For measuring 'unithood', mutual information, log likelihood, t-test, and the notion of 'modifiability' and its variants have been employed. Measures for 'termhood' are limited to frequency-based approaches and the use of reference corpora: the classic TF-IDF (term frequency-inverse document frequency); the notion of 'weirdness' as introduced in [32], which compares term frequency in the corpus with its frequency in a reference corpus; and measures such as 'domain pertinence' in [33] and 'domain specificity' in [34]. The trend in more recent research is to use hybrid approaches, in which 'unithood' and 'termhood' are combined to produce an unified indicator, such as 'C-value' [35] and many others [33,36]. A comparison of different multiword term recognition methods is available in [18].

In the life sciences, there has been a growing recognition of the need for term recognition over the past ten years, although most of the focus has been on entity recognition (i.e. recognising gene names, proteins, etc., cf. especially the Biocreative Challenge [37]). A survey of term recognition for the life sciences is provided by [38], and there has been continuing work leading to the availability of online tools for term recognition such as Termine  that implements a version of the 'C-value' algorithm [35].

As noted below, the selection of fundamental units is a key step in the building of an ontology or any controlled vocabulary. Afzal et al. [39] used term recognition as a basic step in building a controlled vocabulary, while [12] used a set of seed terms to identify relevant terms and built a controlled vocabulary for the Habitat domain. The use of seed terms or seed ontologies has also been used for ontology building in general [8,40]. The standard NLP approach to ontology learning is, however, to use a domain corpus and derive the ontology from the terms present in that collection [9,41].

### Distribution of terms within journal sections

Our investigation of whence animal behaviour terms arise within the different section of the corpus was interesting, with the *Title *and *Abstract *shown to be enriched with animal behaviour terms; the *Materials and Methods *and *Results *relatively poor in terms, and the *Introduction *and *Discussion *having levels of animal behaviour terms similar to the average for the whole corpus.

The result that the 'title only' *Bibliography *is very much enriched with animal behaviour terms was initially a surprise. Upon reflection, however, such a result is easily explained. A research paper prioritises *new *information over *given *information [42,43]. Any one research article on animal behaviour will tend to focus upon a single behaviour, mentioned in the *Title*, and describe this in light of other behaviours via citation. It is taken for granted that the reader of a research article on animal behaviour is an expert in animal behaviour – thus little background is given. As the subject matter of the research is traditionally mentioned in an article's title, a collection of such titles is an excellent source of terms for that area. Similarly, abstracts, being shorter than other sections, are also relatively rich in behavioural terms.

However, the title of cited references are even shorter and are likely to mention the same behaviours that are mentioned in the abstracts of those articles. Thus, when looking for terms that are the subject matter of the journal, we can make a tentative recommendation that considerable progress could be made simply to download all the titles of journal articles, if the full text corpus was not available.

### Term recognition and ontology focussing

A key challenge in the process of learning an ontology from texts is to identify the base units, i.e. the set of terms that will be used as labels in the ontology's class hierarchy. This problem has been largely ignored in the NLP ontology learning literature. There is an implicit assumption that, for example, when an ontology for travel is required, such an ontology will be built from a 'travel corpus' (see, for example [44]). A corpus on travel, however, contains much information outside the scope of travel. The problem of constructing an ontology from a corpus such as the one we were using is that there are, in effect, a number of different domain ontologies represented in the text. In the journal *Animal Behaviour*, there exist terms reflecting experimental methods, animal names, other named entities (places, organisations, people), etc., in addition to behaviours (see Figure 2). Such domains are obviously pertinent to animal behaviour (e.g. there are species-specific behaviours), but the terms relevant to those domains belong in separate ontologies. The linking together of concepts from such separate domain ontologies is a further step in the process of ontology building [24], and our work shows that the ability to *focus *initially upon the terms appropriate for a particular ontology is of the highest importance in this kind of approach.

### Improving the text processing methodology

Our particular approach, striving towards high recall at the expense of precision, produces artefacts with many useful terms but much noise. The limitations of our approach may be summarised as follows:

1. There is a large amount of noise in the resulting ontologies.

2. Some effort is involved in focussing the created ontology to exclude terms that properly belong to other domains or ontology modules.

3. The result is only taxonomic – the use of string inclusion implies an ***IS_A ***hierarchy although careful inspection shows that this is not always the case.

The most pressing issue is to maintain or improve the moderately good recall while increasing the poor precision to a level where the 'good terms' are not overwhelmed by the 'bad'. The artefacts produced by the current process are far from formal ontologies, but nonetheless is certainly useful as a step towards a taxonomic hierarchy for the annotation of research objects, and as a stepping-stone to a more formal ontology.

### The cost and benefit of a text processing approach to ontology construction

#### (a) Time

It is difficult to quantify the resources used in producing the ontology-like structures presented in this paper. Considerable human effort was used during the evaluation steps (manually determining recall and precision, for example), over and above that needed for the generation of the ontologies themselves. Excluding that, we reckon the following as parts of the human effort required to produce the outputs was as follows:

• Preparation of corpus – adapting files into a format that can be processed: 8 hours;

• Preparation and application of regular expression for animal behaviour terms: 4 hours;

• Setting up various automation processes: 2 hours.

As such, in terms of human time, the actual resources required to create the artefacts were low, if we omit the obvious time taken for inspection of the ontologies that any ontologist would employ as part of good practise.

In contrast to this low level of input of human resource, the computer resource used was high. The application of five different automatic term recognition algorithms took several days of processing. Furthermore, the use of the lexico-syntactic pattern to check subsumption relationships against the Web took many days. However, while the computational time for such an approach is long, it is time during which a human can undertake other activities.

#### (b) Quality

The assessment of cost-benefit is somewhat more difficult. While the production of these ontology-like artefacts was at low cost, these were, at least in the early stages, full of irrelevant, poor material. Initial precisions of 26% using the regular expression method (5a, Table 6) and of 14% using the voting method (5b, Table 6) means that the overwhelming majority of the initial content is 'bad' – a standard phenomenon of natural language processing. Furthermore, the regular expression term extraction method failed to capture more than one quarter of relevant terms from the corpus.

Application of our subsequent processing steps to these initial ontologies (Step 6: String inclusion; Step 7: filtered as to whether top level terms were 'activity, behaviour or action'; and Step 8: re-ordering of the top-level hierarchy) gave considerable improvements, increasing the proportion of top-level terms relevant to animal behaviour to above 40% for both methods (8a and 7b, Table 8), while grouping valuable sub-classes together under these 'index' terms.

The learnt ontologies did, however, also contain errors in terms of structure. When a term is purely semantically compositional, the string inclusion methodology works, but in a substantial minority of cases, for example when a more idiomatic structure is employed, the technique breaks down and poor subsumption relationships are created. For example, female mate selection can reliably break down to mate selection and thence selection. However, the algorithm fails with risk taking behaviour, which *should *be broken down into risk taking and behaviour, and even here the subsumption relationship between the two elements is not straight forward.

Finally, from an ontological perspective, our learnt ontology-like artefacts are not yet well-formed. The ontologies conform to few, if any, of the guidelines proposed by, for example, the Open Biomedical Ontologies consortium [16]. The highly compositional terms suggest an implicit series of ontologies of more atomic concepts as advocated by the approach of normalisation [24] and prototyped here for the concept defence. In addition, the original ABO makes a distinction between behaviour acts and behavioural functions, namely the human interpretations of the purposes of those acts. Thus, for example, a pattern of physical activity such as running is an indisputable behavioural act, but correct classification of its behavioural function (e.g. prey capture or predator avoidance) may depend upon detailed cognitive interpretation. No such distinctions are made in the work presented here. Similarly, no ontological distinction is made as to where any term might appear underneath some upper ontology category such as continuant, occurant or sub-categories thereof. Making such distinctions is an essentially human activity. Although imperfect, the output from our work is of significant ontological usefulness. Some relatively simple human input could rapidly clean up the ontologies: after scanning the top-level terms, the non-behaviour terms could be deleted, along with their sub-trees of similarly bad terms, significantly improving the precision with relatively little effort.

Perhaps the most useful aspect of the generated ontologies is as characterisations of the domain. It is easy to see from the learnt ontologies what is important in the corpus and thus, it is to be hoped, in the domain itself. As a purely subjective observation, the ontologists within the project found this characterisation extremely useful.

## Conclusion

In the introduction, we asked whether it is more appropriate to gather a large number of ontological terms rapidly and then tidy up, or to expand a formal ontology gradually until it reaches a useful size. We have not fully answered this question, but have demonstrated that it is possible to produce a starting point for a large-scale formal ontology-building effort with little cost in terms of human effort, irrespective of whether the ultimate ontology is intended for a specific application (application ontology) or reference (reference ontology) [45].

The significance of our approach is that it can be undertaken with relatively low effort, compared to the traditional approach to ontology construction using domain experts. The results produced are useful, both in themselves as a knowledge discovery exercise in a scientific domain, and as a stepping stone to a more rigourous or formal ontology. The low effort involved in the process means that this type of data collection could be used in all cases when building ontologies from scratch, where appropriate corpora exist.

Furthermore, it is interesting to note that the two term extraction approaches we employed, regular expression filtering and term frequency voting, garnered sets of terms from our corpus that were significantly different from one another, suggesting that subsequent combination of terms from both sources would permit the creation of a richer and more complete formal ontology of animal behaviour. The great advantage of our corpus-based approach in terms of building useful ontologies is that its starting point is the complete collection of terms actually used in the current literature. In contrast, hand-crafted ontologies are perforce limited in scope by the knowledge of the domain experts participating in the ontology creation exercise. This means that the formal ontology resulting from the corpus-based term extraction method is likely to be more comprehensive and more useful for annotation of real research data. It is our intention to continue this work from these encouraging beginnings and to create a revised and expanded formal Animal Behaviour Ontology for use by the ethological community.

We also propose this approach for the purpose of ensuring that ontologies are up to date and current with the evolving terminology of a domain

## Competing interests

The authors declare that they have no competing interests.

## Authors' contributions

The authors collaborated in all aspects of the work reported on in this paper.
